# Study on Creep Mechanical Properties of HTPB Solid Propellant

**DOI:** 10.3390/ma19101951

**Published:** 2026-05-09

**Authors:** Li Jin, Siqi Jia, Ze Zhang, Zicong An, Zhenkun Lu

**Affiliations:** 1 School of Renewable Energy, Inner Mongolia University of Technology, Ordos 017010, China; 19829085901@imut.edu.cn (L.J.);; 2 School of Robot Engineering, Wenzhou University of Technology, Wenzhou 325035, China

**Keywords:** hydroxyl-terminated polybutadiene (HTPB), creep, constitutive model, simulation

## Abstract

Solid rocket propellants based on hydroxyl-terminated polybutadiene (HTPB), in which HTPB acts as the polymeric binder and fuel matrix, are widely used in aerospace propulsion. During storage, transport, and service, these composite energetic materials are exposed to sustained mechanical loads as well as environmental variations, which may induce time-dependent inelastic deformation. Such creep deformation can alter the grain geometry, affect combustion stability, and reduce the structural reliability of rocket motors. In this work, room-temperature tensile creep tests were conducted on an HTPB-based solid propellant under different stress levels. Several viscoelastic and power-law constitutive models were compared, and a composite time-hardening creep model was established to describe the experimental strain–time response. The model was further implemented in Abaqus through a Fortran user subroutine for finite element simulation. The results provide a useful basis for creep deformation assessment, formulation optimization, and structural reliability analysis of HTPB-based propellants.

## 1. Introduction

Solid rocket propellant serves as a critical energy carrier in aerospace applications, with HTPB (Hydroxyl-Terminated Polybutadiene) propellant being one of the most widely used fuel formulations [1]. Throughout its lifecycle—including storage, transportation, and operation—the propellant is subjected to various factors such as temperature fluctuations, pressure changes, and mechanical loads, all of which can induce physical and chemical alterations [2]. Under prolonged mechanical loading, the propellant may experience creep, a time-dependent inelastic deformation that results in gradual shape change. This phenomenon can adversely affect rocket performance, structural stability, and operational safety [3]. If creep behavior is not accurately predicted and controlled, it may lead to structural failure or performance deviations, influencing critical parameters such as thrust and burn rate, and potentially compromising mission success. Hence, a precise creep constitutive model is essential for providing a theoretical foundation for rocket design and performance optimization, ensuring reliable operation under diverse conditions.

The study of creep mechanical properties in solid propellants represents a significant research focus, particularly concerning material behavior under high temperature, high pressure, and multiaxial stress states [4,5,6]. In real-world applications such as rocket motors and missile systems, propellants are frequently exposed to extreme environments where sustained loads can lead to progressive creep deformation. Since strain builds up over time and not instantly, understanding creep is crucial to guarantee performance stability for the mission’s entire duration [7,8,9].

Investigations into creep properties are directly linked to the service life evaluation and durability assessment of solid propellants [10]. Under practical conditions, creep-induced deformation may impair the functional integrity and safety of the propulsion system [11,12]. A thorough comprehension of creep mechanisms is therefore vital for ensuring system reliability [13]. Moreover, creep data inform the structural design of crucial components such as combustion chambers and nozzles, supporting material selection and adaptation to demanding mission profiles which including extended orbital operations and high-temperature environments [14].

Current experimental methodologies for studying creep are generally divided into two categories: constant-stress tests, where the specimen is loaded until fracture while strain evolution is recorded, and constant-strain tests, where stress relaxation is monitored until failure [15,16,17,18,19]. Since the latter often does not result in complete fracture, stress-controlled tests are more commonly employed in creep studies. Typical creep behavior under constant load is characterized by a three-stage strain–time curve, each stage corresponding to different underlying deformation mechanisms [20,21].

Research approaches primarily combine experimental testing, numerical simulation, and theoretical modeling. Experimental techniques including tensile [22], compressive [23], thermal creep [24], and high-strain-rate tests are used to investigate creep mechanisms at multiple scales. For instance, microstructural analyses have revealed that environmental conditions significantly influence creep behavior through mechanisms such as particle–matrix debonding. Macroscopically, studies have demonstrated a logarithmic-linear relationship between applied stress and time-to-creep-failure, with some materials exhibiting multi-stage creep even under elastic loading.

Numerical simulations often utilize finite element analysis (FEA) or computational fluid dynamics (CFD) to model the mechanical response of propellants under complex loading scenarios [20,21,25,26,27,28]. For example, viscoelastic models based on the Wiechert [29] formulation have been developed to accurately capture creep compliance, showing good agreement with experimental data.

Theoretical efforts focus on constitutive modeling using empirical and mechanistic approaches to formulate creep equations. Established models such as the Norton [30] power law, generalized Kelvin [31] model, and Schapery’s [32] viscoelastic formulation are commonly applied. Multi-parameter viscoelastic models have proven particularly effective in fitting experimental data, with certain parameters exhibiting minimal temperature dependence.

For engineering applications, these findings must be integrated into the design process of rocket propulsion systems [33]. Appropriate material selection and structural design strategies are essential to mitigate creep effects, ensuring operational reliability under variable thermo-mechanical conditions [34]. A multidisciplinary approach combining testing, modeling, and simulation is necessary to fully characterize creep behavior and meet the stringent requirements of advanced aerospace systems [35].

As a key constituent of solid propellants, HTPB significantly influences overall propulsion performance and reliability. In-depth research into the creep behavior of HTPB-based propellants under sustained load provides insight into material characteristics and deformation mechanisms, thereby supporting optimized formulation and processing techniques. Given the complex, nonlinear nature of creep, accurate prediction remains a challenge for researchers and engineers. Developing robust constitutive models can significantly enhance predictive accuracy and improve the understanding and control of propellant deformation.

Accordingly, this paper presents an experimental investigation into the creep mechanical properties of HTPB solid propellants. A constitutive model describing their creep behavior will be established, and a corresponding user subroutine will be developed to simulate and predict creep response under various loading conditions.

## 2. Experiment

### 2.1. Material

The test material was an HTPB-based solid rocket propellant. The propellant consisted of AP (ammonium perchlorate, 69.50 wt.%) as the oxidizer, Al powder (18.50 wt.%) as the metal filler, DOS (di-2-ethylhexyl sebacate, 3.40 wt.%) as the plasticizer, MAPO (tri-(2-methylaziridinyl) phosphine oxide, 0.05–0.10 wt.%) as the bonding agent, TDI (2,4-toluene diisocyanate, 1.0–2.0 wt.%) as the curing agent, HTPB (hydroxyl-terminated polybutadiene, 0.6–0.7 wt.%) as the polymeric binder, and liquid additives (0.50–1.0 wt.%). The larger AP particles had diameters of 100–400 µm, while the smaller AP particles were less than 35 µm. The aluminum powder had a median particle size of D50 = 22 µm. According to the supplier specification, the Al particles were approximately near-spherical, and their particle-size grade was consistent with commercial 325-mesh aluminum powder. These constituents formed a highly filled crosslinked HTPB matrix after curing.

The curing system was based on TDI. Because the specimens were obtained from a cured propellant batch, the exact supplier, molecular-weight distribution, degree of polymerization of the HTPB prepolymer, and crosslink density of the cured network were not fully recorded in the available batch documentation. This limitation is acknowledged, and future work will include systematic characterization of HTPB molecular parameters and crosslink density to strengthen the interpretation of creep mechanisms.

### 2.2. Sample Preparation

**Figure 1:**
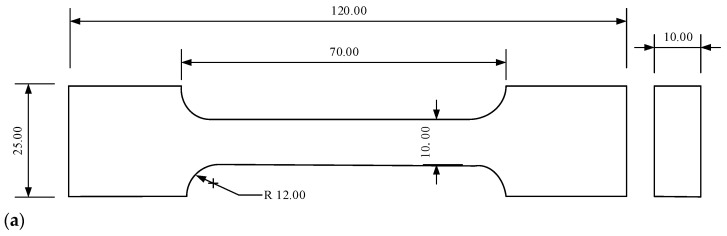

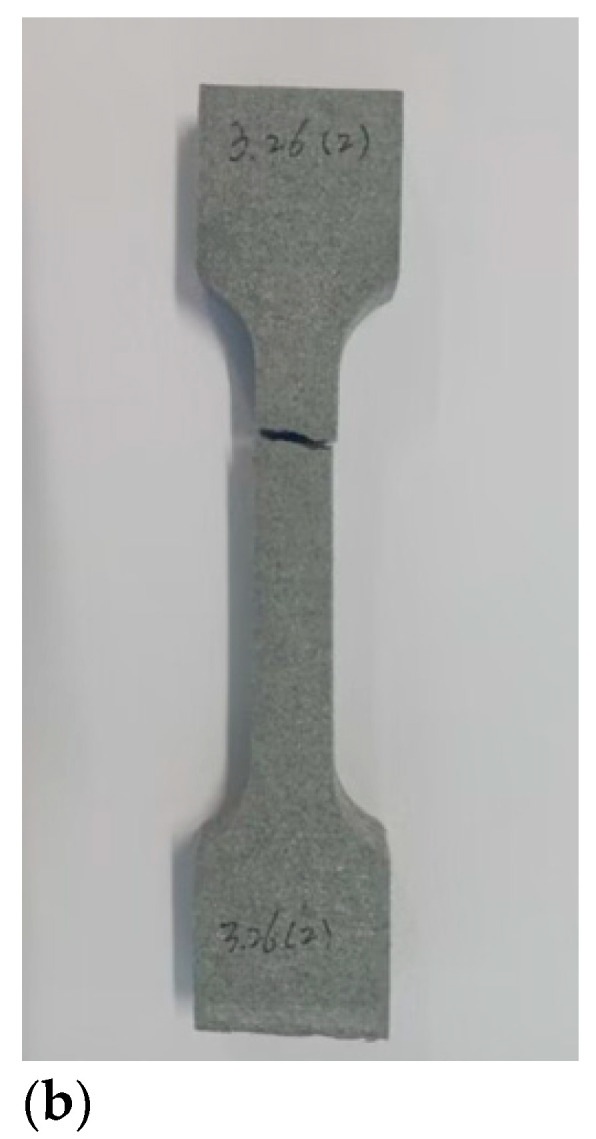
Geometry and appearance of the HTPB propellant uniaxial tensile specimen: (**a**) schematic of the dumbbell-shaped specimen designed according to QJ 924-85; (**b**) physical specimen used for tensile and creep tests.

According to the Chinese aerospace industry standard, QJ 924-85, the uniaxial tensile sample was designed as a dumbbell shape, as shown in Figure 1.

### 2.3. Experimental Device

**Figure 2:**
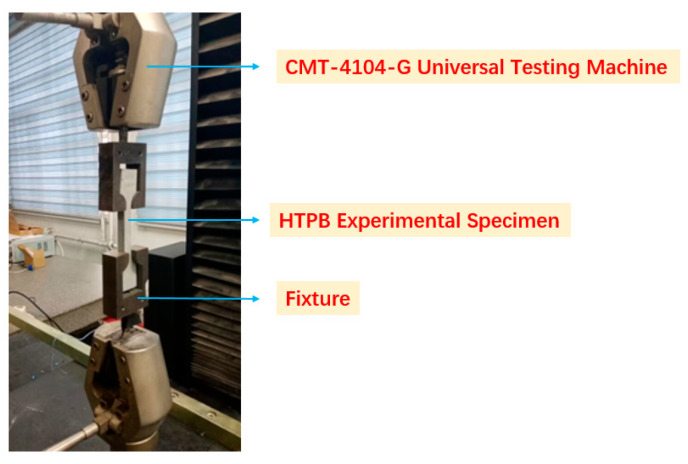
Uniaxial tensile instruments and specimen.

The uniaxial tensile and tensile creep tests were carried out on a testing machine (CMT-4104-G, Shenzhen SANS Testing Machine Co., Ltd., Shenzhen, China) at an ambient laboratory temperature of approximately 23 °C, as shown in Figure 2. The temperature was monitored as the nominal room temperature during testing; however, the detailed temperature-control accuracy was not continuously logged. This point is considered a limitation because the viscoelastic response of HTPB-based propellants is temperature-sensitive.

### 2.4. Experimental Methods

**Figure 3:**
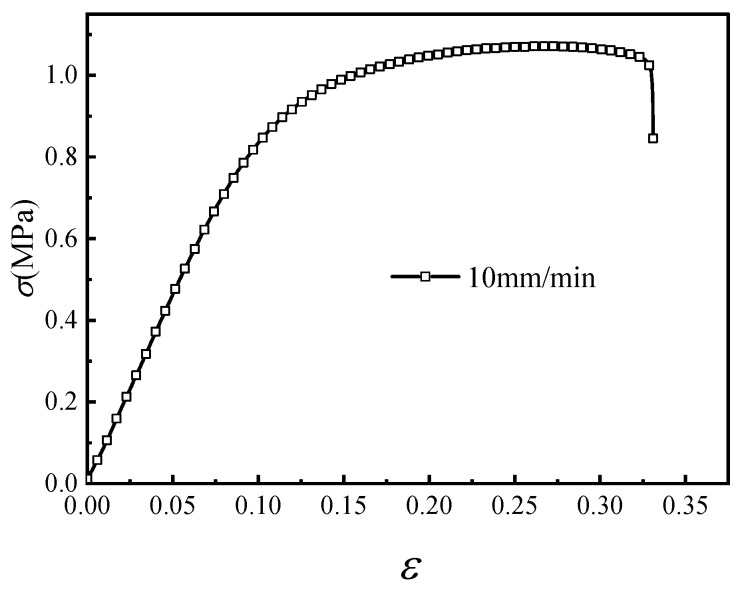
Engineering stress–strain curves at a stretch rate of 10 mm/min.

The stress levels for creep testing were selected according to the uniaxial tensile response obtained at a stretching rate of 10 mm/min (Figure 3). The maximum tensile strength was approximately 1.1 MPa. Therefore, the calibration creep tests were conducted at 0.1, 0.2, 0.4, 0.5, 0.55, 0.6, 0.75, 0.8, 0.85, and 1.0 MPa, covering the main stress range before tensile failure. Additional creep tests at 0.45 and 0.9 MPa were reserved for model validation rather than parameter calibration. For each stress level, the reported curve corresponds to one valid specimen. A complete replicate-based statistical analysis, including standard deviation and error bars, was not available for the present batch; this limitation is explicitly acknowledged in the revised discussion.

The creep test control method chosen in this paper is to control the stress level. The specific steps are as follows:

(1) Specimen inspection: ensure that the geometry and size of the HTPB solid propellant specimen meet the specifications.
(2) Device setting: Set the control parameters of CMT-4104-G testing machine, mainly set the stress level and test duration.
(3) Starting the test: Start the testing machine and make it operate at the predetermined temperature and stress level. The test duration is usually long and the creep behavior of the material is observed during the test.
(4) Data recording and analysis: During the test, the creep strain of the specimen is observed over time. When the test reaches a predetermined duration, stop the testing machine.
(5) Summarize: Summarize the results of the creep test and write a report.

## 3. Results and Discussions

### 3.1. Test Results and Data Processing

The tensile force applied to the specimen and the displacement produced by the specimen during the test can be read directly from the testing machine. Therefore, it is necessary to convert the tension and displacement collected during the test into the stress and strain values we need. In this paper, in order to eliminate the cutting errors caused during specimen preparation, the dimensions of the specimen to be subjected to creep test are carefully measured with vernier calipers and the average of the measured values is taken as the initial cross-sectional area of the specimen. The calculation equations of engineering stress and engineering strain during the test are as follows:

(1) $$\sigma =\frac{F}{A_{0}}$$

(2) $$\varepsilon =\frac{L-L_{0}}{L_{0}}$$

where $A_{0}$—initial cross-sectional area of the specimen, in mm^2^; $L_{0}$—initial standard length of the specimen, in mm.

**Figure 4:**
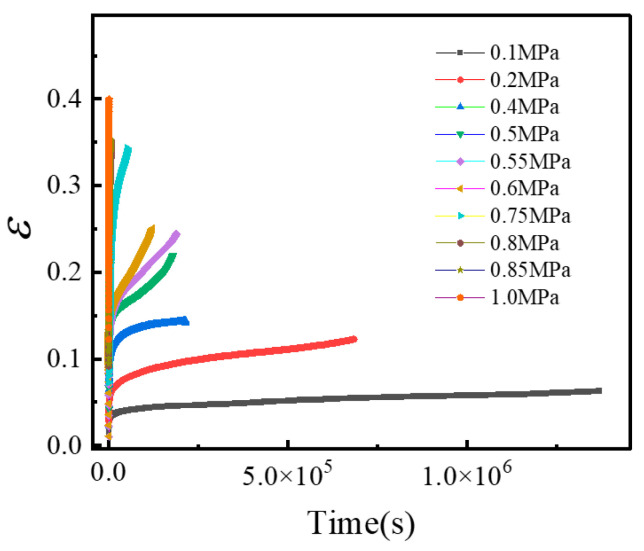
Creep test strain–time curve.

Based on Equations (1) and (2), the strain–time curve of creep test under different stress levels can be obtained as shown in Figure 4.

**Figure 5:**
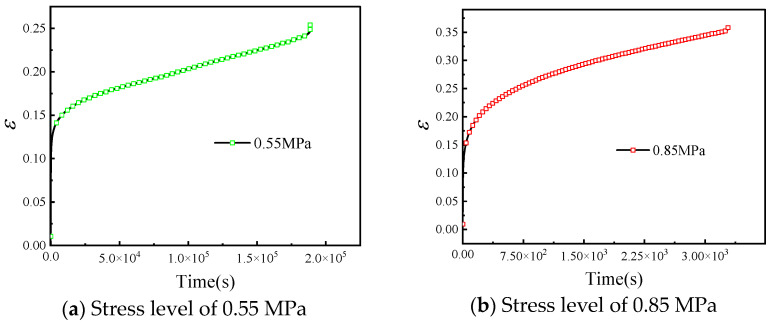
Representative engineering strain–time creep curves of HTPB propellant under two stress levels: (**a**) 0.55 MPa and (**b**) 0.85 MPa. The ordinate represents engineering strain, which is dimensionless (mm/mm), and the abscissa represents time (s).

Taking the HTPB solid propellant creep test results of 0.55 MPa and 0.85 MPa there in as an example, the strain time curves are shown in Figure 5.

As shown in Figure 5, the strain–time curves of HTPB solid propellant exhibit similar stage characteristics under different stress levels. Immediately after loading, an instantaneous strain appears because of the elastic response of the matrix and the particle-filled network. The subsequent primary creep stage is characterized by a gradually decreasing strain rate. With continued loading, the material enters a secondary creep stage in which the strain rate is relatively stable. At high stress levels or long loading times, the strain rate increases markedly, corresponding to tertiary creep and progressive damage accumulation before failure. Thus, the creep response can be described by three stages: decelerating creep, approximately constant-rate creep, and accelerating creep.

### 3.2. Computational Framework

HTPB solid propellant is a highly filled polymer-bonded composite with pronounced viscoelastic and time-dependent mechanical behavior. Its creep response is governed by the elastic deformation of the polymer network, viscous flow of the binder phase, particle–binder interfacial interaction, and progressive damage such as particle debonding or microvoid growth under sustained tensile stress.

The viscoelastic properties and creep properties are closely related. A higher effective elastic modulus and stronger particle–binder bonding generally reduce the initial strain and creep compliance, whereas increased binder mobility, excessive plasticizer migration, or weak interfacial bonding may increase the creep strain. Therefore, constitutive modeling should describe both the initial elastic response and the time-dependent strain evolution under sustained loading.

To identify a suitable creep constitutive model for HTPB solid propellant, five spring-dashpot viscoelastic models and three power-law-type creep models were evaluated. The viscoelastic models included the Kelvin model, the three-parameter solid model, the three-parameter fluid model, the four-parameter solid model, and the four-parameter fluid model. The power-law-type models included the time-hardening model, the composite time-hardening model, and the Schapery model.

**Figure 6:**
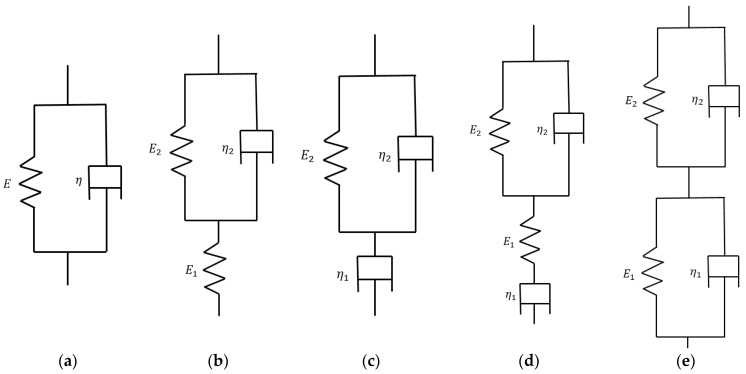
Combination of spring-loaded sticky pots for five viscoelastic ontological models. (**a**) Kelvin model; (**b**) Three-parameter solid model; (**c**) Three-parameter fluid model; (**d**) Four-parameter fluid model; (**e**) Four-parameter solid model.

As can be seen from Figure 6, the Kelvin viscoelastic model is a combination of a viscous pot and a spring in parallel. Its creep equation is as follows:

(3) $$\varepsilon (t)=(\frac{1}{E}-\frac{e^{-\frac{E}{\eta }t}}{E})\sigma_{i}$$

The three-parameter solid viscoelastic model is based on the Kelvin viscoelastic model combined with a spring in series. Its creep equation is as follows:

(4) $$\varepsilon (t)=(\frac{1}{E_{1}}+\frac{1-e^{-\frac{E_{2}}{\eta }t}}{E_{2}})\sigma_{i}$$

The three-parameter fluid viscoelastic model, on the other hand, is based on the Kelvin viscoelastic model combined with a viscous pot in series. Its creep equation is as follows:

(5) $$\varepsilon (t)=(\frac{t}{\eta_{1}}+\frac{1-e^{-\frac{E_{2}}{\eta_{2}}t}}{E_{2}})\sigma_{i}$$

The creep equation for the four-parameter fluid viscoelastic model is given below:

(6) $$\varepsilon (t)=(\frac{1}{E_{1}}+\frac{t}{\eta_{1}}+\frac{1-e^{-\frac{E_{2}}{\eta_{2}}t}}{E_{2}})\sigma_{i}$$

The four-parameter solid viscoelastic model can effectively describe the nonlinear viscoelastic behavior of materials by connecting two Kelvin viscoelastic models in series, which is especially suitable for complex materials such as biological tissues and polymers. Its creep equation is as follows:

(7) $$\varepsilon (t)=(\frac{1-e^{-\frac{E_{1}}{\eta_{1}}t}}{E_{1}}+\frac{1-e^{-\frac{E_{2}}{\eta_{2}}t}}{E_{2}})\sigma_{i}$$

where $\sigma_{i}$ is the constant engineering stress applied during the test (MPa); ε(t) is the engineering strain measured at time *t*; *E*_1_, *E*_2_, and *E*_3_ are the elastic moduli of the spring elements (MPa); and η, $\eta_{1}$ and $\eta_{2}$, are the viscosity coefficients of the dashpot elements.

The power rate class models selected in this paper are: time-hardening eigenmodel, composite time-hardening eigenmodel and Schapery eigenmodel. Among them, the creep equation of the time-hardening eigenstructure model is:

(8) $$\varepsilon (t)=At^{n}$$

The creep equation for the composite time-hardening eigenstructure model is:

(9) $$\varepsilon (t)=At^{n}+Bt$$

The creep equation for the Schapery eigenmodel is:

(10) $$\varepsilon (t)=A+Bt^{n}$$

where *A*, *B*, and *n* are the parameters to be fitted for the power-law-type creep model.

**Figure 7:**
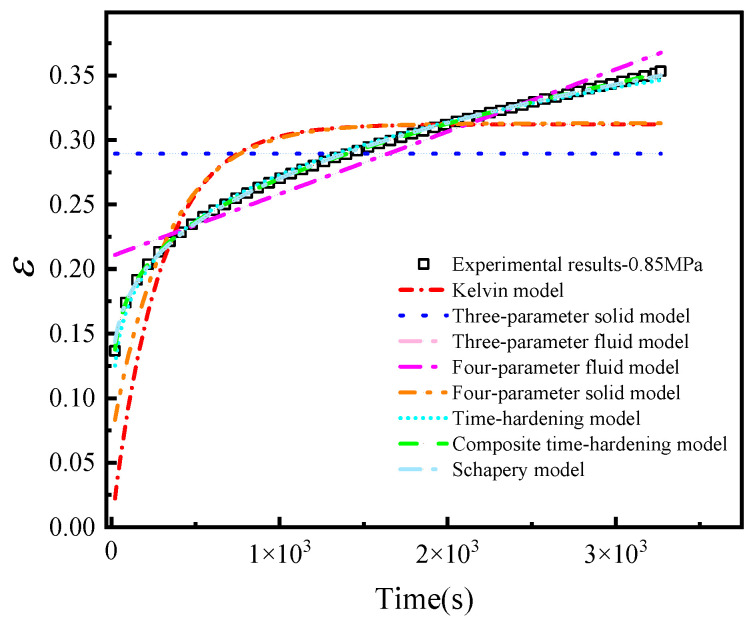
Fitting results for the 0.85 MPa creep curve using the candidate constitutive models. The ordinate represents engineering strain, which is dimensionless (mm/mm), and the abscissa represents time (s).

The creep test results of HTPB solid propellant at a stress level of 0.85 MPa were fitted using nonlinear least-squares regression. The objective function minimized the sum of squared residuals between the experimental engineering strain and the model-predicted strain over the full time range. The coefficient of determination (R^2^) and root mean square error (RMSE) were used as quantitative criteria for model comparison, where $R^{2}=1-\frac{\sum_{i=1}^{m}{\left(\sigma_{i}-{\hat{\sigma }}_{i}\right)}^{2}}{\sum_{i=1}^{m}{\left(\sigma_{i}-\bar{\sigma }\right)}^{2}}$. In the equation, $\sigma_{i}$ represents the parameter values identified at each stress level in Table 1, ${\hat{\sigma }}_{i}$ represents the values calculated using the corresponding fitting equation, and $\bar{\sigma }$ represents the average of the experimental parameter values. The closer *R*^2^ is to 1, the better the fitting equation represents the trends in the experimental data. The fitting results are shown in Figure 7.

As shown in Figure 7, the five spring-dashpot viscoelastic models have limited ability to reproduce the full creep process of HTPB solid propellant, particularly the nonlinear strain evolution at later loading times. In contrast, the three power-law-type models describe the time-dependent creep response more effectively.

**Figure 8:**
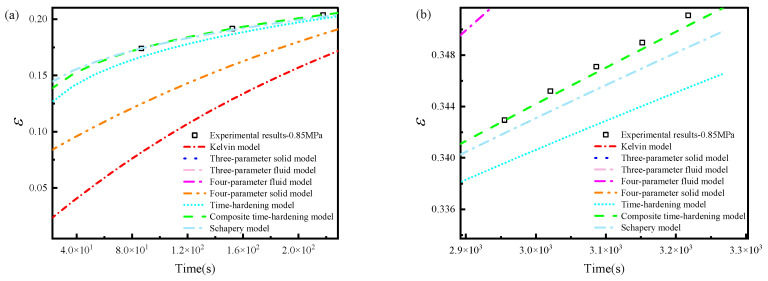
Local enlarged views of the fitted 0.85 MPa creep curve: (**a**) primary creep stage; (**b**) accelerating creep stage. The ordinate represents engineering strain (dimensionless, mm/mm).

The fitting results of the three power-law-type models were further compared by locally magnifying the primary creep stage and the accelerating creep stage, as shown in Figure 8. The composite time-hardening model provided the most balanced description of both the initial creep response and the late-stage strain acceleration. Therefore, this model was selected for subsequent parameter identification and finite element implementation.

### 3.3. Fitting of Creep Test Curves

**Figure 9:**
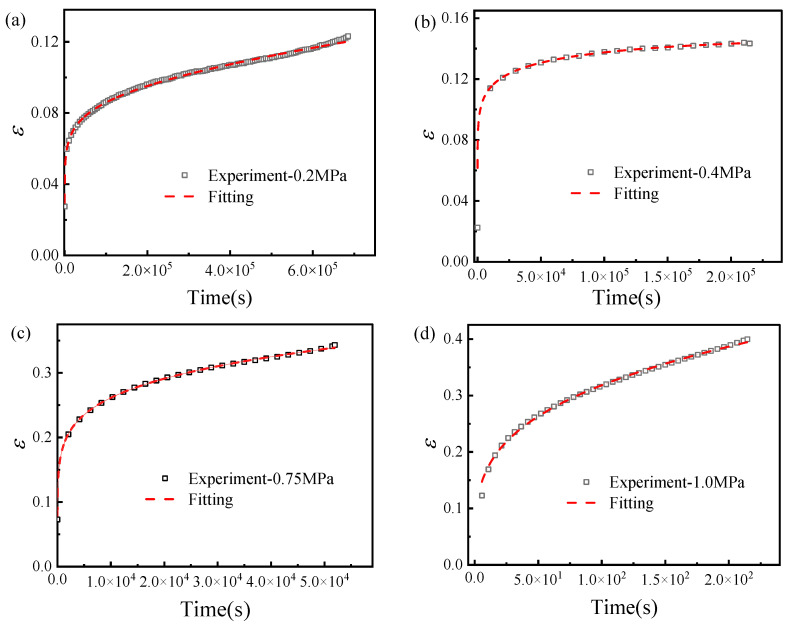
Comparison between experimental and fitted engineering strain–time curves at different stress levels: (**a**) at a stress level of 0.2 MPa; (**b**) at a stress level of 0.4 MPa; (**c**) at a stress level of 0.75 MPa; (**d**) at a stress level of 1.0 MPa. The ordinate represents engineering strain (dimensionless, mm/mm), and the abscissa represents time (s).

The creep test curves at the calibration stress levels were fitted using Equation (9) by nonlinear least-squares regression, and the comparison between experimental and fitted curves is shown in Figure 9. The fitted parameters A, B, and n of the composite time-hardening model are listed in Table 1.

### 3.4. Establishment of Creep Constitutive Model

**Figure 10:**
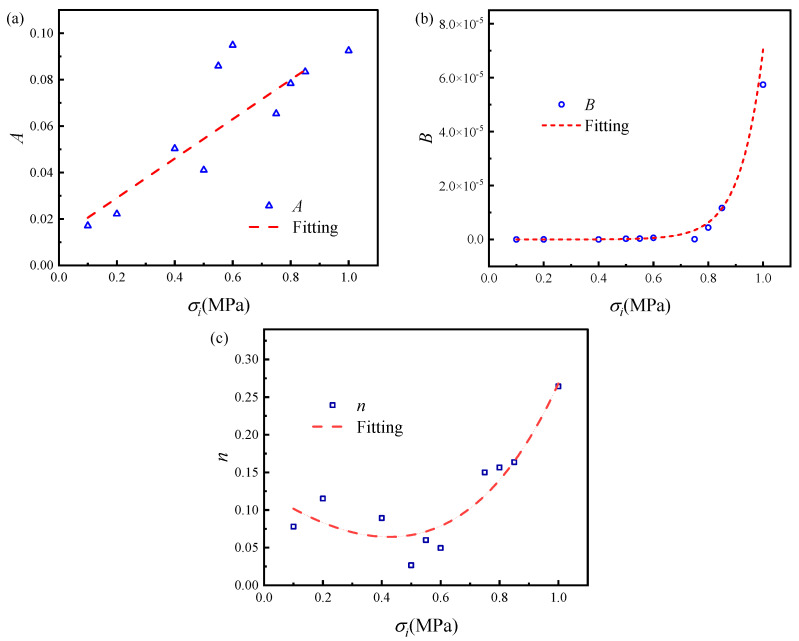
Stress dependence of the fitted parameters *A*, *B*, and *n* in the composite time-hardening model: (**a**) *A*-stress relationship; (**b**) *B*-stress relationship; (**c**) *n*-stress relationship. *A* and *n* are dimensionless parameters, while *B* has units consistent with the time-dependent term in Equation (9).

Based on the fitting results in Table 1, the stress dependence of the three parameters in the composite time-hardening model was analyzed, as shown in Figure 10. The fitting equations corresponding to the parameters in Figure 10, the coefficients of determination for parameters *A*, *B*, and *n* were 0.8138, 0.9362, and 0.8652, respectively. The results indicate that parameter *B* exhibits the best fit with respect to stress levels, followed by parameters *A* and *n*. Overall, the established parameter-stress relationship effectively reflects the variation in the composite time-hardening constitutive model parameters with respect to stress levels, with parameter *B* demonstrating the highest fitting accuracy.These stress-dependent parameter functions were then substituted into the composite time-hardening equation to establish a stress-dependent creep constitutive model.

The physical meanings of the three parameters are as follows. Parameter A mainly reflects the initial strain component associated with the instantaneous elastic response and early-stage deformation after loading. Parameter B controls the magnitude of the time-dependent creep strain and can be regarded as a creep compliance-related coefficient. Parameter n governs the time sensitivity and nonlinear evolution rate of creep strain. The increase in B and n at higher stress levels indicates stronger time-dependent deformation and possible damage accumulation in the particle-filled HTPB matrix.

(11) $$A=0.0849\sigma_{i}+0.01207$$

(12) $$B=4.089\times 10^{-10}\;e^{12.05\sigma_{i}}$$

(13) $$n=0.125e^{1.7846\sigma_{i}}-0.4766\sigma_{i}$$

In summary, the creep equation for the composite time-hardened form of HTPB solid propellant is given as:

(14) $$\varepsilon (t)=(0.0849\sigma_{i}+0.01207)t^{0.125e^{1.7846\sigma_{i}}-0.4766\sigma_{i}}+(4.089\times 10^{-10}\;e^{12.05\sigma_{i}})t$$

**Figure 11:**
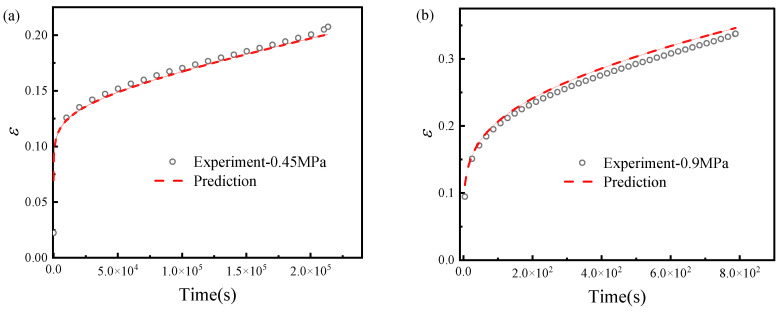
Validation of predicted and experimental engineering strain–time curves: (**a**) at 0.45 MPa and (**b**) at 0.9 MPa. The ordinate represents engineering strain (dimensionless, mm/mm), and the abscissa represents time (s).

To verify the predictive capability of the creep equation, Equation (14) was used to predict the creep test results at 0.45 MPa and 0.9 MPa. These two stress levels were not used in parameter calibration; 0.45 MPa represents an intermediate interpolation case, and 0.9 MPa represents a relatively high-stress validation case close to the failure range. As shown in Figure 11, the predicted curves agree reasonably with the experimental curves, indicating that the proposed constitutive model can characterize the tensile creep response of HTPB solid propellant within the investigated stress range. Broader validation under additional stress levels, temperatures, and loading modes will be conducted in future work.

**Figure 12:**
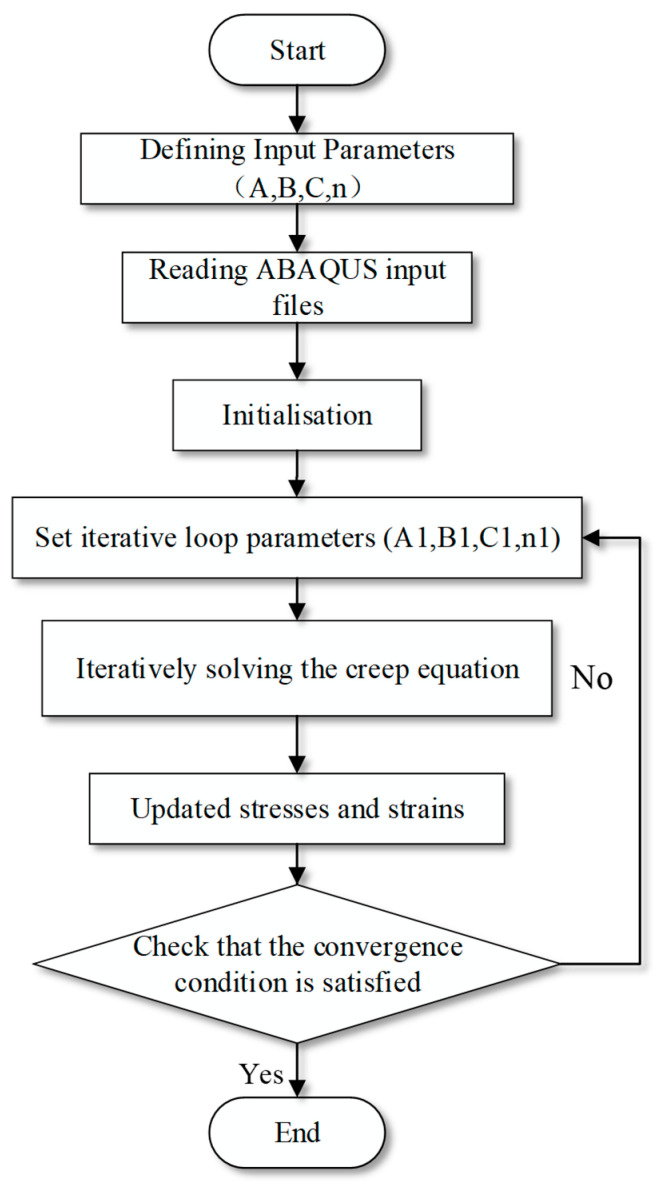
Flow chart of the Fortran user subroutine used to implement the composite time-hardening creep model in Abaqus.

Finite element simulations were carried out using Abaqus/Standard. The stress-dependent composite time-hardening creep model in Equation (14) was implemented through a Fortran user material subroutine. The elastic modulus used in the initial loading step was obtained from the initial slope of the uniaxial tensile stress–strain curve in Figure 3 and was taken as 7.868 MPa. The creep parameters A, B, and n were calibrated from the experimental creep curves and then expressed as functions of stress according to Equations (11)–(13). The flow chart of the user subroutine is shown in Figure 12.

**Figure 13:**
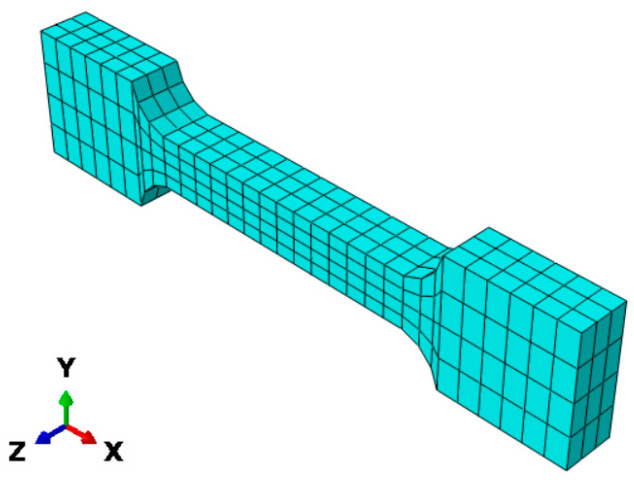
HTPB solid propellant finite element model.

A dumbbell-shaped finite element model was established according to the specimen geometry shown in Figure 1, as illustrated in Figure 13. Because HTPB propellant exhibits rubber-like near-incompressible behavior, the incompressible hexahedral hybrid element C3D8H was selected. A systematic mesh-independence study was not performed in the present work; therefore, the potential influence of mesh size is recognized as a limitation and will be evaluated in future simulations.

**Figure 14:**
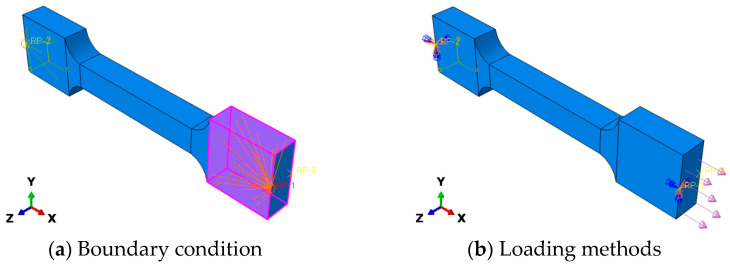
Boundary condition and loading setup of the finite element model: (**a**) coupling boundary condition applied to both specimen ends; (**b**) tensile loading method with one end fixed and the other end subjected to constant stress.

The boundary condition and loading scheme are shown in Figure 14. Coupling constraints were applied to both ends of the specimen to keep the end sections flat and to prevent rigid-body rotation during loading. One end of the specimen was fixed, while a constant tensile stress was applied at the other end, consistent with the experimental creep loading method.

**Figure 15:**
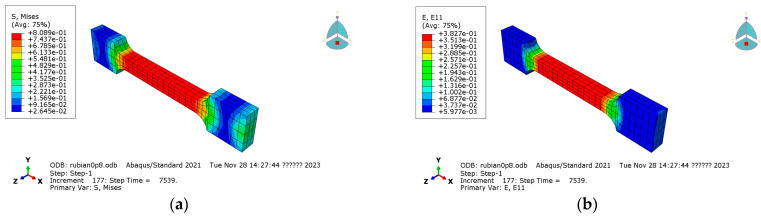
Finite element contours calculated for the 0.8 MPa case: (**a**) Mises stress contour in the tensile direction; (**b**) tensile strain contour.

**Figure 16:**
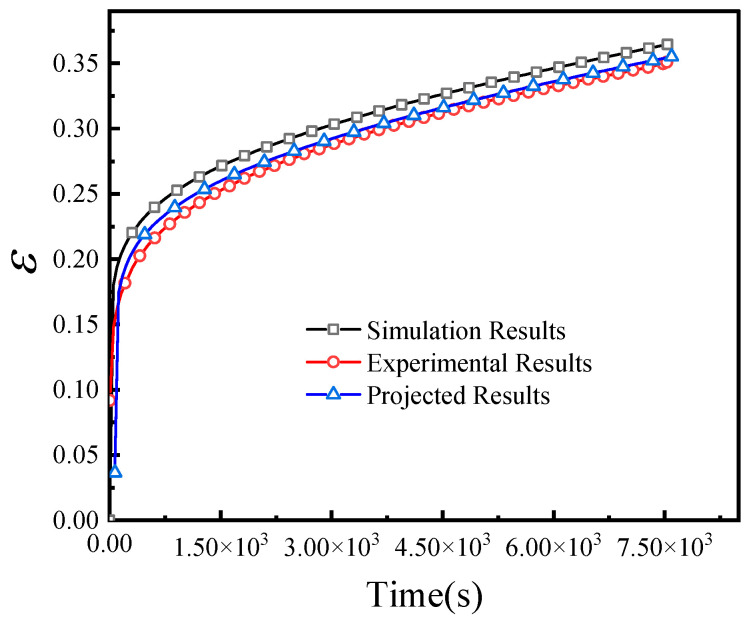
Comparison among finite element simulation, experimental result, and constitutive-model prediction at the 0.8 MPa stress level. The ordinate represents engineering strain (dimensionless, mm/mm), and the abscissa represents time (s).

**Figure 17:**
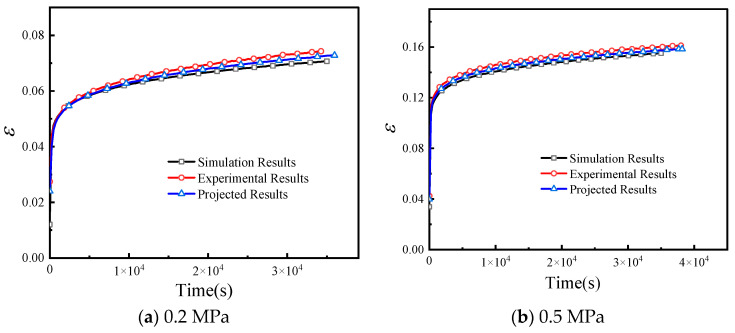
Comparison among finite element simulation, experimental result, and constitutive-model prediction at two stress levels: (**a**) 0.2 MPa; (**b**) 0.5 MPa. The ordinate represents engineering strain (dimensionless, mm/mm), and the abscissa represents time (s).

Taking the 0.8 MPa case as an example, the Mises stress contour and tensile strain contour calculated by the user subroutine are shown in Figure 15. A representative node in the middle section of the specimen was selected for comparison with the experimental curve and the constitutive-model prediction, as shown in Figure 16. The predicted curve and experimental curve show good agreement. The finite element curve is slightly higher than the experimental and analytical prediction curves, which is mainly attributed to the initial elastic loading stage and the sensitivity of the simulation to the elastic modulus. Additional simulations at 0.2 MPa and 0.5 MPa were also carried out for the first 35,000 s, and the results are shown in Figure 17.

In summary, the user subroutine based on the composite time-hardening model can simulate and predict the tensile creep behavior of HTPB solid propellant under the investigated stress levels. In engineering applications, the model can be used as a material input for preliminary rocket motor grain structural analysis, allowing engineers to estimate long-term deformation, identify high-risk stress regions, and compare the creep resistance of candidate formulations under sustained mechanical loading.

### 3.5. Practical Implications, Creep-Resistance Improvement, and Limitations

The creep resistance of HTPB-based propellants may be improved through both formulation and processing strategies. Possible approaches include optimizing the AP/Al particle-size distribution to increase packing density, improving particle–binder interfacial bonding with bonding agents or surface treatments, adjusting the NCO/OH ratio and curing conditions to obtain a more stable crosslinked network, reducing excessive plasticizer migration, and introducing compatible reinforcing fillers such as nano-silica or carbon-based additives at appropriate contents. These measures should be balanced against processability, combustion performance, and safety requirements.

Several limitations of the present work should be noted. First, SEM or CT microstructural characterization was not conducted because of the safety restrictions associated with cutting, polishing, and imaging energetic propellant samples; therefore, the discussion of particle distribution and interface-related mechanisms is based on macroscopic creep behavior rather than direct microscopic evidence. Second, only one valid creep curve at each stress level was used for model calibration, and full statistical variability, including standard deviations and error bars, was not available. Third, the tests were performed at approximately 23 °C, and the temperature-control accuracy was not continuously recorded. Fourth, the current model was validated only at two additional stress levels and one temperature, and a systematic mesh-independence analysis for the finite element model was not performed. Future work will include safe microstructural characterization, replicate tests, multi-temperature creep experiments, and mesh-sensitivity analysis to further improve the reliability and generality of the model.

## 4. Conclusions

In this study, tensile creep tests were conducted on HTPB solid propellant under different stress levels, and the corresponding engineering strain–time curves were obtained. Several viscoelastic and power-law-type constitutive models were compared, and a stress-dependent composite time-hardening creep model was established. The model was implemented in Abaqus through a Fortran user subroutine to simulate the creep behavior of the propellant. The main conclusions are as follows:

(1) The creep behavior of HTPB solid propellant material follows a pattern similar to traditional materials, comprising three distinct stages: decelerating creep, constant-rate creep, and accelerating creep.
(2) Five spring-dashpot viscoelastic models and three power-law-type creep models were used to fit the 0.85 MPa creep curve. Based on fitting accuracy and the ability to capture both the primary and accelerating creep stages, the composite time-hardening model showed the best overall applicability.
(3) By fitting the creep curves at different stress levels, the stress-dependent relationships of parameters A, B, and n were obtained. The resulting composite time-hardening constitutive equation can predict the tensile creep response of HTPB solid propellant within the investigated stress range.
(4) The Fortran user subroutine developed in this study reproduced the uniaxial creep behavior of HTPB solid propellant with reasonable agreement between finite element simulation, constitutive-model prediction, and experimental results. The proposed model can provide a reference for preliminary creep assessment and structural reliability analysis of HTPB-based rocket motor grains.

## Figures and Tables

**Table 1:** Fitting results for the three parameters in the composite time-hardened ontological model.

Stress (MPa)	*A*	*B*	*n*
0.1	0.01712	7.68 × 10^−9^	0.0780
0.2	0.02217	2.30 × 10^−8^	0.1153
0.4	0.05035	−3.26 × 10^−10^	0.0893
0.5	0.01107	3.14 × 10^−7^	0.0267
0.55	0.08592	3.28 × 10^−7^	0.0601
0.6	0.09489	6.19 × 10^−7^	0.0496
0.75	0.06537	1.12 × 10^−7^	0.1500
0.8	0.07843	4.39 × 10^−6^	0.1565
0.85	0.08342	1.17 × 10^−5^	0.1636
1	0.09253	5.74 × 10^−5^	0.2642

## Data Availability

The original contributions presented in this study are included in the article. Further inquiries can be directed to the corresponding author.
